# Structure-Function Based Molecular Relationships in Ewing's Sarcoma 

**DOI:** 10.1155/2015/798426

**Published:** 2015-01-22

**Authors:** Roumiana Todorova

**Affiliations:** Institute of Biophysics and Biomedical Engineering, Bulgarian Academy of Sciences, Acad. G. Bonchev Strasse, Building 21, 1113 Sofia, Bulgaria

## Abstract

Ewing's Sarcoma Oncogene (*ews*) on chromosome 22q12 is encoding a ubiquitously expressed RNA-binding protein (EWS) with unknown function that is target of tumor-specific chromosomal translocations in Ewing's sarcoma family of tumors. A model of transcription complex was proposed in which the heterodimer Rpb4/7 binds to EAD, connecting it to Core RNA Pol II. The DNA-binding domain, provided by EFP, is bound to the promoter. Rpb4/7 binds RNA, stabilizing the transcription complex. The complex Rpb4/7 can stabilize the preinitiation complexes by converting the conformation of RNA Pol II. EWS may change its conformation, so that NTD becomes accessible. Two different mechanisms of interaction between EWS and RNA Pol II are proposed: (I) an intermolecular EWS-EWS interaction between two molecules, pushing conformation from “closed” to “open” state, or (II) an intramolecular interaction inside the molecule of EWS, pushing conformation of the molecule from “closed” to “open” state. The modified forms of EWS may interact with Pol II subunits hsRpb5 and hsRpb7. The EWS and EFPs binding partners are described schematically in a model, an attempt to link the transcription with the splicing. The proposed model helps to understand the functional molecular interactions in cancer, to find new partners and ways to treat cancer.

## 1. Introduction

The chromosomal translocations that result in the fusion of the amino transactivation domain of TET proteins with the DNA-binding domain of ETS-related transcription factor proteins are the common determinants of cancer [1]. Ewing's sarcoma family of tumors (ESFTs) is an example of how genome research has advanced the understanding of the molecular pathogenesis of the disease. Modulation of EWS/FLI1 expression is a therapeutic goal that may influence the course of the disease [2]. The clarification of the mechanism of EWS function may help to understand the functional molecular interactions in cancer, to find new partners and ways to treat cancer.

### 1.1. EWS

Ewing's Sarcoma Oncogene on chromosome 22q12 is encoding a ubiquitously expressed RNA-binding protein, the Ewing's sarcoma (EWS) protein, a member of the TET (TLS/EWS/TAF15) family of RNA- and DNA-binding proteins.

The EWS protein is an oncogenic RNA-binding protein with MW of 68,478 Da and Basal Ip of 9.37. EWS is consisting of a strong N-terminal transcriptional activation domain (EWS-Activation-Domain (EAD), NTD, and amino acids (AAs) 1–264) and a C-terminal RNA-binding domain (RBD, CTD), extensively methylated at arginine residues and containing RNA-binding motif and a putative zinc-finger domain [3]. The EAD is intrinsically disordered resembling many chromatin organizing proteins [4]. Multiple tyrosine residues are essential for EAD function [4]. Potential molecular recognition features are tyrosine-dependent and correlate well with EAD function, while phenylalanine can effectively substitute for tyrosine. The EAD activates transcription strongly* in vitro* and the effect of EAD mutations is strikingly different from that observed* in vivo* [5].

The cellular role of the normal EWS protein is not well characterized. The exact mechanism of EWS participation in the multiple levels of gene expression is not defined as well as the role of EWS in the pathogenesis of the resulting cancers [6]. The normal EWS is active in both normal tissues and tumor cells [7]. EWS is a multifunctional protein acting in transcriptional coactivation, DNA-recombination, DNA pairing and DNA-repair, splicing, and mRNA transport. EWS protein is located in the nucleus, associated with components of the basal transcription, RNA-splicing factors, and G-protein coupled receptor signaling [8, 9]. The EWS role in amyotrophic lateral sclerosis (ALS) and in other neurological diseases still remains unveiled [10]. EWS is essential for early brown fat lineage determination [11].

### 1.2. EFPs

The EWS gene is target of tumor-specific chromosomal translocations in Ewing's sarcoma family of tumours, myxoid liposarcoma, malignant melanoma of soft parts, desmoplastic small round cell tumor, small round cell sarcoma, acute leukaemia, extraskeletal myxoid chondrosarcoma, and others (Table 1). EFPs (EWS fusion proteins) are potent transcriptional activators that interact with other proteins required for mRNA biogenesis and induce tumorigenesis by perturbing gene expression, due to the EAD and a DNA-binding domain from the fusion partner. The generation of chromosomal translocations in Ewing's sarcoma could be mediated by a mechanism of illegitimate recombination before interchromosomal joining [12]. The most frequent translocation in ESFTs is the EWSR1/FLI-1 translocation t(11;22)(q24;q12) leading to different isoforms, composed of the NTD of EWS (chromosome 22), fused in frame to the CTD of FLI (chromosome 11). The fusion gene can vary depending on whether exons 5–9 or 6–9 of FLI-1 are included. A transcription-independent contribution of the EAD was proposed on the basis of deletion analysis and mutagenesis on EWS/FLI-1 DNA [13, 14]. Both EWS/FLI-1 and FLI-1 act as antiapoptotic agents, targeting the CBP/p300 pathways* in vitro* and* in vivo* [15]. EWS/FLI1 includes the regions EAD (1–265 AAs), RGG1, FLI-1 (37 AA) at the translocation break point (TBP), FLI-1 DNA-binding domain (85 AA), and FLI-1 CTD (89 AA). Wild-type FLI-1 contains a weak TAD in its N-terminus and an ETS DNA-binding domain in its C-terminus.

The translocation t(12;22)(q13;q12) is leading to the fusion EWS/ATF1 that contains the NTD of EWS (chromosome 22), fused in frame to the C-terminus of ATF1 (chromosome 12). The EWS/ATF1 fusion protein in soft tissue clear cell sarcoma is composed of the EAD (residues 1–325), fused to the C-terminal region of ATF1 (residues 66–271). EWS/ATF1 is a potent constitutive activator of the ATF-dependent promoters, dependent on the EAD and the bZIP domain (AA 214–271), consisting of a basic region that directly contacts DNA, and a leucine zipper (ZIP) from ATF1. Wild-type ATF1 contains a weak TAD in its N-terminus and a DNA-binding domain in its C-terminus.

The “alternate” EWS-based fusions, including EWS/ZSG, EWS/NFATc2, EWS/POU5F1, EWS/SMARCA5, and EWS/SP3, may not bind and regulate the same set of target genes as EWS/FLI, as well as the other TET/ETS fusions [16]. EWS/ZSG isoforms are composed of the NTD (EAD) of EWS, fused to CTD of ZSG, and contain a Zn finger at C-terminus, originated from the ZSG.

## 2. Methods

The Intrinsic Protein Disorder (IPD) was predicted by several Predictors, including IUPred [17], DisEMBL [18], RONN [19], and PONDR [20]. Predictors are used for estimation of IPD of EWS isoform 2 (656 amino acids (AA)) [Homo sapiens] (NP_005234.1) and isoforms of EWS oncogenic proteins EWS/FLI1 (476 AA; AAK11227.1), EWS/ATF1 (432 AA; ADX41458.1), and EWS/ZSG long B isoform (609 AA; AAG09037.1). Several reported experimental data were analyzed to propose a mechanism and to build a model of the transcription activation by the EAD.

## 3. Results and Discussion

### 3.1. Intrinsic Disorder

Intrinsically disordered proteins (IDPs) and regions (IDRs) lack stable structure and are linked to the function in signaling, regulation, and control. Proteins associated with human diseases, such as cancer, are enriched in intrinsic disorder: they enter in high-specificity-low-affinity interactions and one-to-many binding mode by which a single IDP/IDR binds to multiple structurally diverse partners, accomplished by their plasticity [21]. Many types of DNA-binding domains in Transcription Factors (TFs) are well structured and specifically recognize DNA; others are highly unstructured and undergo a disorder-to-order transition upon binding to specific DNA [22]. A high level of intrinsic structural disorder is enabling fusion proteins to evade cellular surveillance mechanisms. The translocation-related human proteins are enriched in disorder (43.3% versus 20.7% in all human proteins) and their translocation breakpoints tend to avoid domain splitting. The vicinity of the breakpoint in the oncogenic fusions is significantly more disordered. The structural disorder is essential to the oncogenic function [4, 23].

**Figure 1:**
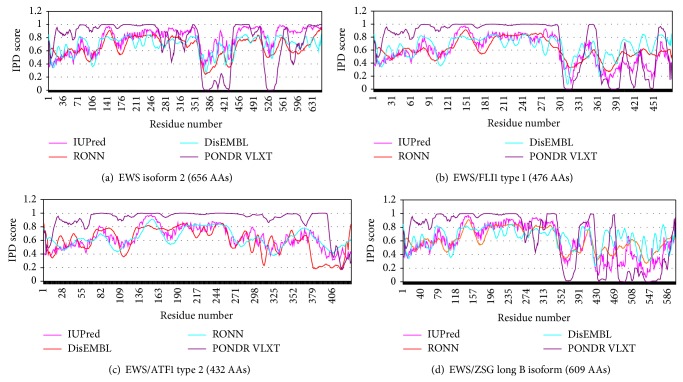
The IPD of native protein EWS isoform 2 (656 AAs) and isoforms of EWS oncogenic proteins EWS/ FLI1 (476 AA), EWS/ATF1 (432 AA), and EWS/ZSG long B isoform (609 AA) were estimated by Predictors IUPred [3], DisEMBL [5], RONN [7], and PONDR [8]. Higher IPD score is equivalent to higher disorder tendency estimated by the Predictor. (a) EWS isoform 2 (656 AAs). (b) EWS/FLI1 type 1 (476 AAs). (c) EWS/ATF1 type 2 (432 AAs). (d) EWS/ZSG long B isoform (609 AAs).

The IPD of the protooncoprotein EWS and of the oncogenic fusion proteins EWS/FlI1, EWS/ATF1, and EWS/ZSG (Figures 1(a), 1(b), 1(c), and 1(d)) was estimated by the Predictors IUPred [17], DisEMBL [18], RONN [19], and PONDR [20].

#### 3.1.1. EWS w.t. (1–656)

By all Predictors the NTD of EWS consists of large disordered regions, while the CTD is almost completely disordered. The N-terminal activation domain (AA 1–264) (EAD) of EWS is almost completely disordered, including also the linking region and big portions of the CTD. Small amounts of short partially ordered regions with no globular domains were shown within the whole EWS molecule (Figure 1(a)). The EAD is involved in self-association and oligomerization. The interaction mechanism involves the highly disordered N-terminal and centrally localized AAs, while for optimal association the full-length molecule is required. An RNA component is involved in the EWS oligomerization [24], found also to be relatively disordered.

#### 3.1.2. EFPs

*(1) EWS/FLI1*. EWS/FLI1 was shown to approach a largely unfolded conformation under native conditions [25]. By all Predictors the NTD (EAD, AA 1–264) of the fusion protein EWS/FLI1 shows high intrinsic disorder as the same domain of native EWS (Figure 1(b)). The CTD of the fusion, originated from FLI1, is almost ordered and shows relatively low propensity for disorder. The predictions of three oncogenic fusions with different isoforms of Fli1 show different disorder propensity. The globular domains in all EWS/FLI1 isoforms are disposed closely to the FLI1 C-terminus of the fusion, but not at the C-terminal end of the molecule. The EWS/FLI1 self-associates and binds to FLI1 via its C-terminal DNA-binding domain [24] that has relatively low tendency for disorder.

*(2) EWS/ATF1*. The EWS fusions show similar intrinsic disorder in the NTD (AAs 1–264) with the same domain of native EWS (Figure 1(c)) by all Predictors. Two different EWS/ATF1 isoforms were estimated for intrinsic disorder that give different results. An increased disorder showed the regions, flanking the bZIP domain, while the rest of the CTD is almost ordered. The bZIP domain is folded and linked by highly conserved sequences that are mobile and unstructured. The critical elements and particularly the position of the breakpoint are connected by long segments of structural disorder. The calculated distance/disorder between the oncogenic elements TAD and b-ZIP was of 280/265 AAs [23]. A globular sequence is situated at the C-terminus of the molecule (in the bZIP domain of ATF1, AA 214–271) that mediates dimerization and DNA-binding.

*(3) EWS/ZSG*. EWS/ZSG is a Zing-finger type oncogenic protein in “Ewing's-like” tumors. The IPD of EWS/ZSG, estimated by all Predictors, showed a long disordered region, originated from EWS, followed by a globular domain in the CTD, comprising the A–T hook DNA-binding motif and Zn finger, and a short relatively disordered region at the C-terminal end (Figure 1(d)).

#### 3.1.3. Intrinsic Disorder and Function

The results from different Predictors were compared for native EWS protein and its oncogenic fusion proteins. The EWS fusions, estimated by all Predictors, showed similar intrinsic disorder in the EAD, derived from the native EWS. The decreased IPD in CTD of EFPs is due to the fused TF. A relation between structure, disorder, and function was found in some regions of the studied EWS fusion proteins. The analysis, based on the IPD prediction results for the functional regions of EWS and its oncogenic fusions, allowed making a relationship between IPD and sequence function [26, 27].

The disordered regions were used to generate Abs against EWS, EWS/Fli1, and EWS/ATF1 fusion proteins (free or flexible AA regions for recognition of the target protein). Thus the disordered region of AAs (136–152) in the EAD was used to generate Ab against the EWS protein. The common structural features which are limited to the TET-family members suggest that they bind RNA and/or ssDNA in a unique way [28]. To recognize the EWS/FLI1 protein, an antibody was raised against a peptide, corresponding to AAs (434–452) mapping at the carboxy terminus of the FLI1 protein. The epitope is localized closer to the C-terminus, compared to the ETS binding domain, which is essential for its binding to DNA [29]. To recognize EWS/ATF1, an antibody was raised against the recombinant ATF-1 protein (AAs 137–237). The epitope is localized in part in a relatively disordered region, including a partially globular region of ATF1 with length of about 100 AAs [30].

EAD-target binding is driven probably by a balance between EAD conformational entropy and favorable EAD-target cation-*π* contacts [31].

### 3.2. Summary of Functional Studies on EWS and EFPs

#### 3.2.1. Post-Translational Modifications

The arginine methylation within the RBD of EWS protein is not needed for its subcellular localization and protein-protein interactions. Only methylated EWS protein could be found in the nucleus or at the cell surface of the eukaryotic cells [32]. The recombinant EWS protein is methylated immediately after translation or even cotranslationally in the cytosol of HEK cells by methyltransferase PRMT1. The methylation is affecting its activation/repression activity, RNA-binding properties, and stabilization. Both unmethylated and methylated EWS proteins interact with RNase-sensitive protein complexes, including the RNA-helicases p68 and p72, and relocate from the nucleoplasm to the nucleolar periphery, when coexpressing [33].

TLS/FUS and EWS were identified in mRNA-transporting granules in dendrites of neurons [34]. PRMT8 is expressed particularly in the brain tissue and is localized at the plasma membrane [35]. PRMT8 interacts directly via the RGG box 3 (the preferred methylation-motif within the RNA-binding domain) with the EWS protein, and their colocalization in HEK cells indicates a physiological relevance.

TET-family proteins interact with the cytoplasmic kinase v-Src [36] and are found to localize at the cell membrane, but their function and mechanism are still unknown [37].

The EWS protein relocates from cytoplasm to ribosomes upon Pyk2 activation [8]. The cytoplasmic protein tyrosine kinase Pyk2 is involved in calcium-induced regulation of ion channels and activation of the map kinase signaling pathway. The glycosylation is related to transcriptional activation, cell growth, and link with IGF-1 signaling.

Phosphorylation, acetylation, and glycosylation are posttranslationally modifying mechanisms, affecting EWS/FLI1 activity. EWS/FLI1 is regulated by acetylation [38]. ESFT cells express the histone acetyl transferases PCAF, CBP, and p300. The CTD of EWS/FLI1 is acetylated by CBP, PCAF, and P300: four lysines are major sites for acetylation (K240, K252, K380, and K397), thus modulating its function. Full-length EWS/FLI1 directly binds to both PCAF and P300 and can be acetylated* in vitro* [38]. The retained NH_2_-terminal SYQG domain of EWSR1 functions as a transcriptional activator [39] and could involve changes in histone acetylation status [40]. Thus the post-translational modifications in the EFPs may be altered compared to native TFs.

#### 3.2.2. Self-Association of EWS and EFPs

The EWS is ubiquitously expressed RNA chaperone that all three (RGG) motifs participate in self-associating, required for the EWS nuclear import [14]. The interaction mechanism of EWS involves the N-terminal and centrally localized amino acids, while for optimal association full-length EWS molecules are required [24].

Self-association of EWS and EWS/FLI1 (but not FLI1) and interaction of EWS/FLI1 with EWS and FLI1 were observed* in vivo*. The EWS NTD, present in both EWS and EWS/FLI1, contributed to homo- and heterotypic interactions. The EWS/FLI1 was able to self-associate and bind to FLI1 via its CTD, a DNA-binding motif [24]. Involvement of an RNA component in EWS oligomerization was confirmed by sensitivity to RNaseA treatment. The formation of inactive homo- or heterodimers could be a general mode of regulating Transcription Factors activity* in vivo* [41].

The EWS/WT1 is phosphorylated* in vivo* on Ser and Tyr, thus affecting the DNA-binding and homodimerization. The tyrosine phosphorylation of EWS/WT1 by c-Abl negatively regulates its DNA-binding properties [42]. The binding of several EWS/WT1 molecules leads to homotypic associations that translate into transcriptional effects [43]. The NTD of EWS/WT1 shares homology with the CTD of the large subunit Rpb1 of Pol II that is phosphorylated by c-Abl on 52 Tyr, thus converting transcriptionally paused complexes into elongation competent molecules [42]. The EWS/WT1 self-association is mapped to the fusion junction and is negatively influenced by phosphorylation. DNA-binding and self-association domains overlap, but DNA-binding does not depend on self-association [43].

The EWS/ATF1 fusion protein binds to the ATF sites, present in the cAMP-responsive promoters via the bZIP domain of ATF1, and activates transcription. The transactivation by EWS/ATF1 does not require dimerisation with other ATF family members [44]. The bZIP domain (aa 214 ± 271), consisting of a basic region that directly contacts DNA and a leucine zipper (ZIP) that allows dimerisation, is necessary and sufficient for dimerisation and DNA-binding [44]. The inhibition of B-ZIP TFs could be therapeutically useful in cancer cells, where oncogenesis is driven by a B-ZIP protein, such as in clear cell sarcoma [45].

#### 3.2.3. EWS/FLI1-Mediated Regulation of the Gene Expression

EWS fusion proteins participate in signaling cascades required for oncogenesis [16]. EWS/FLI may have DNA-binding independent function, related to a dominant negative function of EWS/FLI, blocking the normal function of wild-type EWS expression [16]. The very flexible and unfolded conformation, changing its shape, allows EFPs to bind and possess many interacting partners. Direct or indirect target genes of EWS/FLI contribute to various aspects of tumor growth and progression, such as IGFBP3, GSTM4, CDKN1A, TGFBRII, VEGF, CAV1, E2F8, FOXO1, and NFKBIL2 [46, 47]. The “core” regulators, including genes NR0B1, NKX2.2, and GLI1 are absolutely essential for oncogenic transformation in Ewing's sarcoma [16]. EWS/FLI1 repressed miRNAs, targets in IGF signalling pathway, such as miR-100, miR-125b, miR-22, miR-221/222, miR-271, and miR29a. EWS/FLI1 may be able to induce large gene expression changes by causing smaller alterations in multiple stages of gene regulation [35]. EWS/FLI1 can dictate steady state target gene expression by modulating both transcript synthesis and mRNA degradation [35]. EWS/FLI1 alters transcription and RNA stability in ESFT cells. Intact EWS and ETS domains are required for full repression of IGFBP3 by EWS/FLI1 (via decreased transcript half-life of IGFBP3). EWS/FLI1 alters Pol II recruitment at Tsp2 in NIH 3T3 cells [35]. Uridine phosphorylase (Upp) is a direct target gene, necessary for tumorigenesis, upregulated by EWS/FLI1 in NIH 3T3 cells [48]. EWS/FLI1 repressed the expression of direct target genes at the level of transcript synthesis. ChIP experiments showed that EWS/FLI1 decreases the amount of Pol II at the promoter of downregulated genes in both murine and human model systems. Full EWS/FLI1-mediated transcriptional repression requires intact EWS and ETS domains [49].

#### 3.2.4. EWS Partners in Transcription

EWS serves as a bridge between the components of the basal transcriptional machinery [28] and the splicing apparatus [9, 50, 51] of the general gene expression. The common structural features of TET-family members suggest that they bind RNA and/or ssDNA in a unique way [28]. The TET interactions with TFIID, RNA polymerase II (Pol II), and elements of the RNA-splicing machinery indicate a role in transcription and mRNA splicing [49]. The TETs, as classical TFs, are associated with the transcriptional preinitiation complex, with RNA Pol II enzyme, and TFIID complex, thus functioning from the initiation of transcription to the delivery of the mature mRNA to the cytoplasm [1]. The SYGQ-rich transcription-activation domain of TLS (EWS) may bind RNA, RNA Poll II and RPB3, TFIID, and nuclear hormone receptors, while the RNA-binding domain may bind YB-1, NF-kB, TASR1/2, and SC-35 in addition to RNA, ssDNA, and dsDNA [1]. Main protein-protein interactions of native EWS include EWS-NTD (EAD) binding to hsRPB7, U1C, ZFM1, CBP, and PYK2; EWS-CTD (RBD) binding to YB-1, SR proteins, PRMT1, TFIID, and p68 [10].

The RGG domain in the carboxy terminus of EWS is important for the G-quadruplex specific binding, pointing that the functions of EWS and TLS are modulated by specific structures of ncRNAs [52].

#### 3.2.5. EFPs Partners in Transcription

The interaction partners of EWS/FLI1 may differ from those of the native protein EWS.

The EFPs play a role in the tumorigenic process. They may disturb gene expression by mimicking or interfering with the normal function of CTD-Pol II within the transcription initiation complex. The NTD of EWS/WT1 shares homology with CTD of the large subunit Rpb1 of Pol II [42]. The EWS/FLI1 is acting as a strong transcriptional activator and, in contrast to wild-type Fli1, it is a potent transforming agent [2]. The transcriptional complex of EWS/FLI1 includes RNA Pol II, CREB1, and RHA (DHX9) [53, 54]. EWS/FLI1 protein-protein interaction partners in transcription are hsRBP7, RHA, BARD1, C-JUN, SAP1a, and CBP/p300 [55]. EWS/FLI1 functions by binding to normal cellular protein partners in transcription and splicing, similar to virus corruption of normal cellular machinery for virion production [55].

The EWS/FLI1 fusion gene product is thought to affect the expression of cell cycle-regulatory molecules involved in the control of the G1-S transition. G1 cyclins, including cyclin D1 and cyclin E, are upregulated by EWS/FLI1, while CDK inhibitors of the G1-S transitions, p21 and p27, are downregulated. The p53 pathway is also indirectly affected by EWS/FLI1 [56].

Transcriptional influences between EWS/FLI1, CFLAR, MYC, P300, E2F1, RELA, IER3, and FOXO1 nodes were extracted from the literature-based influence network. c-Myc MYCBP is positively induced by EWS/FLI1 in A673 cell line. Transcriptome time-series after EWS/FLI1 silencing were used to identify core modulated genes, and the connections marked are EWS/FLI1 → E2F1 (through cyclin and RB); EWS/FLI1 → E2F2 (EWS/FLI1-IER3-P300); EWS/FLI1-CFLAR (EWS/FLI1 → MYC); EWS/FLI1 → E2F5 (E2F2) [57].

Binary switch model for EWS/FLI mediated transcriptional regulation points that, at directly repressed genes, such as* LOX* and* TGFBR2*, EWS/FLI may preferentially recruit transcriptional repressor complexes, such as the NuRD complex, with its associated HDACs and LSD1, to transcriptionally inhibit gene expression. At directly activated genes, such as* NR0B1* and* GSTM4*, EWS/FLI may preferentially recruit activator complexes to transcriptionally upregulate gene expression [58].

#### 3.2.6. EWS Partners in Splicing

The spliceosome is a ribozyme that uses a two-metal ion mechanism for catalysis [59]. Disruption of the EWS and YB-1 interaction by CPT may alter the local recruitment of the splicing machinery, affecting MDM2 exon splicing [60]. Treatment with the transcription elongation inhibitor camptothecin leads to substantial exon skipping, and a subset of these events were recapitulated by knockdown of EWS or of its interacting protein YB-1 [60]. The EWS protein interacts via its RBD with RNase-sensitive protein complexes, consisting of mainly heterogeneous nuclear ribonucleoproteins (hnRNPs) and RNA helicases. HnRNPs M and U, the RNA-helicases p68 and p72, and also actin and tubulin were found to interact directly with the EWS protein. Coprecipitation experiments with recombinant proteins confirmed the interaction of the EWS protein with p68 via its RBD [37]. EWS depletion results in alternative splicing changes of genes involved in DNA-repair and genotoxic stress signaling, including ABL1, CHEK2, and MAP4 K2. EWS depletion reduces cell viability and proliferation upon UV irradiation by restoring c-ABL expression at the level of posttranscriptional mechanisms of DNA damage response. EWS dissociates from its targets and relocalizes to nucleoli upon UV irradiation. Thus EWS plays a role in DNA damage response and UV light-induced dissociation of EWS from sites of active transcription, in particular from alternatively spliced regions regulated by this protein, and contributes to splicing changes induced by UV light. The molecular mechanisms behind induced DNA damage changes in gene regulation remain poorly understood [61]. A possibility is that introns facilitate efficient mRNA synthesis, perhaps suggesting some sort of splicing-transcription link after all [62].

#### 3.2.7. EFPs Partners in Splicing

EWS/FLI1 functions by binding to cellular partners in transcription and splicing, similar to the cellular machinery corruption by virus for virion production [55].

Y-box binding protein-1 (YB-1) is a conserved eukaryotic translational regulator that is implicated in cancer progression [63]. By regulating overall protein synthesis, YB-1 may act as a stress adaptor protein in Ewing's sarcoma and other tumours through maintenance of ER homeostasis [63]. The YB-1 plays a role in DNA reparation, transcription regulation, splicing, and mRNA translation, thereby participating in many crucial events in cells. Its effect is dependent mostly on its amount, and hence on regulation of its synthesis. YB-1 specifically interacts with the 5′ UTR of its own mRNA within a region of about 100 nucleotides upstream from the start codon [64].

TLS and EWS fusion proteins may contribute to malignant transformation through disruption of RNA splicing, mediated by TLS and EWS-binding proteins, such as YB-1 [50]. Due to interaction with several RNA processing proteins, including the small nuclear ribonucleoprotein (snRNP) U1C [9], the EWS/FLI1 activity has been linked to RNA transcription and splicing [50]. The U1C and SF1 proteins interact with EWS/FLI1 to modulate splicing, while interactions with YB1 and SR inhibit or alter splicing [55]. Additional proteins involved in the EWS/FLI1 spliceosome, not directly binding to EWS/FLI1, are the TASR proteins [65] and YB-1 [50]. The U1C (SNRPC) expression modulates the transactivation activity of EWS/FLI1* in vitro* and* in vivo* via interaction with its NTD [9]. The functional consequences of heterodimerization between EWS/FLI1 and EWS on RNA-splicing have to be investigated [24, 66]. EWS/FLI1 has been shown to interact with the splicing factor U1C, RNA helicase A (RHA), and the hRBP7 subunit of Pol II, which links the protein to splicing and transcription [67].

An alteration of EWS in Ewing's sarcoma alters the dynamics of Pol II over the CCND1 protooncogene encoding cyclin D1, leading to an increase in its transcription and to an alteration of splicing that results in high levels of the oncogenic cyclin D1b splice isoform. The cyclin D1b isoform is highly expressed in Ewing's sarcoma cells and tumors and stimulates Ewing's sarcoma cell growth [68]. Thus, alterations of transcriptional regulators in disease may lead to splicing alterations.

#### 3.2.8. EWS and EFPs Role in miR Processing

The alterations in miR expression in Ewing's sarcoma involve both EWS/ETS oncogenic fusion-dependent and independent mechanisms and contribute to malignant phenotypes. EWS/FLI1 represses some miRs at the transcriptional level, through direct and indirect mechanisms, and likely also transcriptionally activates other miRs. EWS is a component of the Drosha/DGCR8 miR processing complex, and the copy number of the wild-type intact form is reduced in Ewing's sarcomas with EWS-containing fusions. EWS/FLI1 can also interact with EWS, but consequences of this to miR biogenesis are currently unknown. Dicer is upregulated by EWS/FLI1 in Ewing's sarcoma, and Dicer levels impact oncogenesis in other cancers. TARBP2 downregulation in CD133+ Ewing's sarcoma cells results in diminished expression of a number of miRs [69].

#### 3.2.9. RHA Role

The RHA protein is a nuclear DNA/RNA helicase (encoded in humans by the* DHX9* gene) regulating transcription and splicing. RHA was found in a complex with EWS/FLI1 in ESFT cell lines, binding to EWS/FLI1 target gene promoters (including Id2) and enhancing its function as a transcriptional cofactor [53]. The complex formation between CBP/p300 (target gene activation via CREB) and Pol II requires RHA as modulator of transcription that may induce local changes in the chromatin structure [53, 70]. The ESFT cell lines and patient tumors highly expressed RHA, critical for EWS/FLI1 oncogenic function. The EWS/FLI1 specifically binds the RHA fragment (AA 630–1020) and the interruption of this interaction induces apoptosis* in vivo* and* in vitro* in ESFT cells. This represents a potential novel therapeutic strategy against Ewing's sarcoma [53].

The protein EWS is absent in the RHA complex from HEK293 cell, but possibly RHA binds to wild-type EWS in ESFT. The RHA recognizes a unique protein domain that occurs as a result of the fusion between EWS and FLI1 in ESFT and could link EWS/FLI1 to the spliceosome. EWS/FLI1 and EWS share some protein partners such as BARD1 [71] but uniquely bind others, such as YB1 [50]. The RHA may function differently in the complexes with EWS versus EWS/FLI1, leading to oncogenic transformation in the presence of EWS/FLI1 [53, 54].

#### 3.2.10. Animal Model

The Ews (+/−) mice developed normally and were hypersensitive to ionizing radiation. The loss of EWS protein resulted in reduced lamin A/C expression. The EWS is essential in pre-B cell development and meiosis and is involved in cellular senescence, DNA-pairing, and recombination/repair mechanisms [72].

#### 3.2.11. RNA Polymerase II and Heterodimer Rpb4/7 as EWS/FLI1 Partners

RNA Pol II is composed of 12 subunits, Rpb1–Rpb12 [73]. The crystal structures of yeast Pol II revealed a ten-subunit Core that includes the catalytic active site and a two-subunit complex, comprising Rpb4/7 [74]. The Rpb4/7 associates with Core Pol II through the N-terminal ribonucleoprotein-like domain of Rpb7 and the partially ordered N-terminal region of Rpb4. The Rpb4/7 heterodimer functions at the interface of transcriptional and post-transcriptional machinery, playing an important role in transcription, mRNA transport, and DNA-repair. Similar to general TFs, Rpb4/7 aids the assembly of the initiation complex in the promoter region by interacting with both transcription activators and general TFs, including RNA Pol II, TFIIF, and TFIIB [75]. The Rpb4/7 functions extend beyond. The HsRpb7 has a selective role in nuclear extracts, copurifying with EAD/Fli1, but not with Fli1. The hsRpb7 interacts with TFs, involved in cancer development such as EWS [28, 76, 77], and nephroblastoma overexpressed protooncogene, involved in differentiation of several cell types and target of von Hippel-Lindau protein (a potent tumor suppressor) [78]. The introduction of a combination of hsRpb4 and hsRpb7 in yeast cells, lacking Rpb4 and carrying Rpb7, restores the EAD-dependent activation [79].

### 3.3. Experimental Evidences to Build a Model

By summarizing, there are several experimental evidences, concerning the partnership of EWS and the resulting oncogenic fusion proteins. Several complexes might be formed as result of specific protein-protein interactions between EFPs and its partners, related to tumorigenesis, and are important for building a cancer treatment strategy. These consequences, originated directly from the experiments, are as follows.

1. The full-length EWS forms multifunctional complexes (400–1,300 kDa) with Pol II and with TFIID (and hTAFII68) that may be physiologically relevant [28].
2. The full-length EWS (1–656) interacts with Pol II subunits and other TFs, with Pol II via hsRpb3, and with TFIID subunits, TAFII100 (strong) and TAFII55 and TAFII28 (weak). The GST-NTD of EWS (1–333) interacts with Pol II subunits hsRpb7 (moderate) and hsRpb5 (weak) and with TFIID subunits TAFII100 (strong) and TAFII 55, 28 (weak). The N-terminal (1–82) AAs of EWS possesses full transforming activity of EWS. The AAs (1–82) and (1–57) of EWS interact with Pol II. The GST-CTD of EWS ΔNt (341–656) does not interact with Pol II subunits hsRpb3, and interaction is not identified with hsRpb5 and hsRpb7. The CTD of EWS interacts with TFIID subunits, TAFII18 (moderately) and TAF II55 (weak), does not interact with TAFII1, and is not clear with TAFII28 [28]. Thus, the intact EWS binds hsRpb3* in vitro*, but not hRpb5 or hRpb7, whereas the isolated EAD binds hRpb5 and hRpb7, but not hRpb3 [28, 76]. RPB3 Pol II subunit is involved in the regulation of tissue-specific transcription [80].
3. The EAD contains an IQ domain that is phosphorylated by protein kinase C (PKC) and is interacting with calmodulin (CaM) [81]. The region of EWS, interacting with ZFM1 (a transcriptional repressor, identical to splicing factor SF1), is mapped to 37 amino acids within NTD. The EWS/TLS/hTAFII68 proteins are present in distinct TFIID populations, associated with the RNA Pol II holoenzyme and could cooperate with ZFM1 in mRNA processing during transcript elongation [51]. The EAD binds to the coactivator CREB-binding protein, which is implicated in chromatin remodeling.
4. TBP dimerisation inhibits the DNA-binding, thus regulating the TBP-DNA interaction. Formation of inactive homo- or heterodimers could be a general mode of regulating TFs activity* in vivo* [41].
5. The EAD binds directly to Rpb7 by yeast two-hybrid screening, co-immunoprecipitation, and pull-down assays [28, 76, 77, 79]. The Rpb7 and Rpb4 are required for EAD-mediated transactivation in yeast [82]. Overexpression of recombinant hsRPB7 specifically increased the gene activation by EWS-chimeric TFs [76].
6. The majority of EWS/FLI1 is found in low-MW fractions (67–160 kDa). Both TFIID and Pol II complexes have native MW greater than 600 kDa, suggesting that, in contrast to EWS, EWS/FLI1 is not stably associated with TFIID or Pol II [28]. The EWS/FLI1 was not found to co-immunoprecipitate with TFIID complex in Ewing's sarcoma cell lines. All known transcription activators are not stably associated with TFIID or Pol II.
7. The Rpb7 formed direct contacts with Rpb1, Rpb2, and Rpb6, holding them together in a preferred conformation [74, 75]. The conformation of Pol II changes during different stages and Core Pol II may adopt an open configuration, allowing the dsDNA to enter the active-site groove [9]. The Rpb4/7 associates with Core Pol II through the N-terminal ribonucleoprotein-like domain of Rpb7 and the partially ordered N-terminal region of Rpb4 [75].
8. EWS oncogenic fusions may contribute to malignant transformation through disruption of RNA splicing, mediated by YB-1 [50]. U1C and SF1 proteins interact with EWS/FLI1 to modulate splicing; the interactions with YB1 and SR inhibit or alter splicing [55]. The U1C modulates the transactivation activity of EWS/FLI1 by interaction with its NTD [9]. EWS protein and RNA-helicases p68 or p72 co-localize in the nucleus of HEK cells [33]. YB-1 regulates overall protein synthesis and may act as a stress adaptor protein in Ewing's sarcoma [63].
9. The complex formation between CBP and Pol II requires RHA binding [70]. The EWS/FLI1 specifically bounds the RHA AA (630–1020) and both interact with target gene promoters [53].
10. The functional consequences of EWS and EWS/FLI1 hetero-dimerization on RNA-splicing have to be elucidated [66].
11. EWS is a bridge between the basal transcriptional and the splicing machinery of the gene expression [9, 28, 50, 51], from the transcription initiation to the delivery of the mature mRNA to the cytoplasm [1].
12. EWS/FLI may have DNA-binding independent function related to a dominant negative function of EWS/FLI, blocking the normal function of wild-type EWS expression [16].

All reported studies are based predominantly on the oncogenic fusions EWS/FLI1 and EWS/ATF1. The lack of high MW complex, incorporating EWS/FLI1, could be explained by the participation of additional factors in the complex formation* in vivo* that could be altered* in vitro*. The detected binding complex of EWS/FLI1* in vitro*, predominantly by co-immunoprecipitation, could keep only the strongest interactions, remaining under the purification. The exact mechanisms acting* in vivo* are still unclear. Additional factors may be needed for proper action* in vivo*, where a predominant role is played by the EAD, possibly common for EWS and EFPs. The interaction between EAD and Pol II subunits, including the mobile heterodimer Rpb4/7, could be modulated also by additional factors. Thus, the proposed mechanisms and models are schematically trying to summarize the findings about the functioning of EWS (native) and its oncogenic fusions the EFPs.

### 3.4. Proposed Mechanism of Interaction between EWS and RNA Pol II

The full-length protein EWS interacts with RNA Pol II subunits. Two mechanisms of interaction are possible. First, an intramolecular interaction within full-length protein EWS occurs, leading to a particular conformation of EWS, binding with Pol II. Second,* in vitro* interaction between the full-length protein EWS and its CTD occurs, inducing a particular conformation, able to interact with Pol II [28]. Here the fact that the N-terminus of protein EWS is not accessible (or is in protected conformation) in the cell is supposed. This form may bind to Pol II through its subunit hsRpb3, in addition to other specific interactions with certain cellular targets, or after some post-translational modifications. Thus protein EWS may change its conformation so that its NTD becomes accessible. The modified forms of EWS may interact with Pol II subunits hsRpb5 and hsRpb7.

Taking into consideration the reported experimental data, two different mechanisms could be proposed for the interaction between protein EWS and RNA Pol II, depending on the type of interactions that induces the conformational change of EWS:

 (I) an intermolecular EWS-EWS interaction between two molecules, pushing conformation of the molecule from “closed” to “open” state;
 (II) an intramolecular interaction inside the molecule of EWS, pushing conformation of the molecule from “closed” to “open” state.

**Figure 2:**
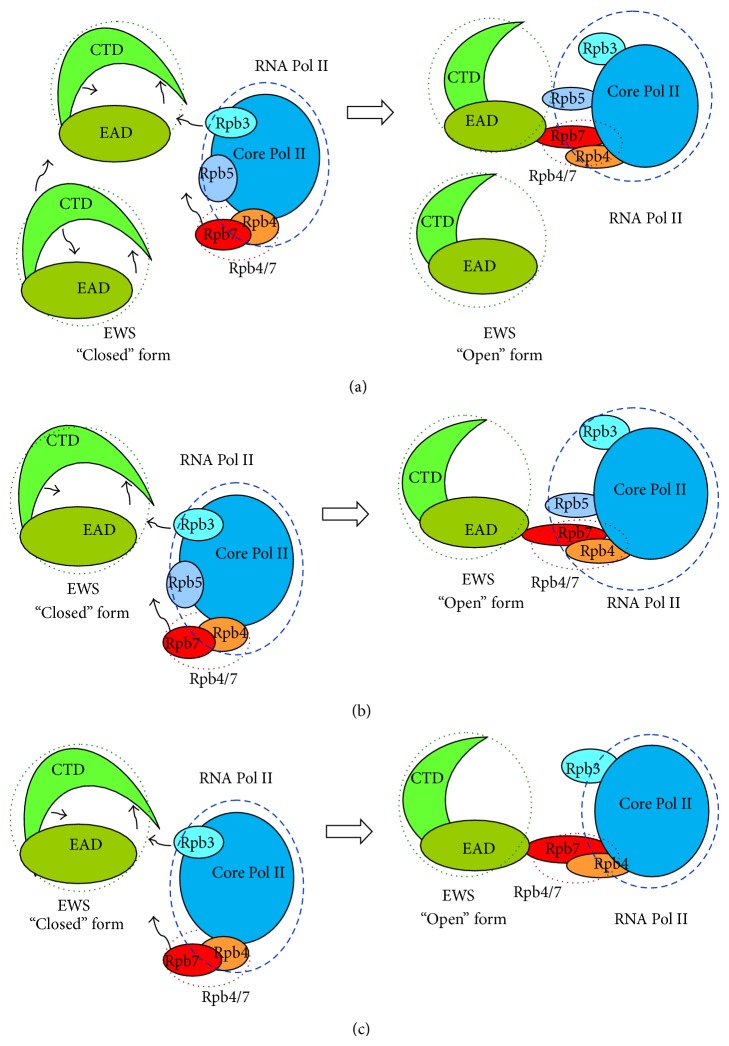
Proposed mechanism of the interaction between EWS and RNA Pol II (presented schematically). (a) Mechanism I. The EWS-EWS intermolecular protein-protein interactions induce the transition from “closed” to “open” conformation of the molecule, thus making the N-terminal part of EWS accessible for interaction with Rpb5 and the heterodimer Rpb4/7. (b) Mechanism II. An intramolecular interaction inside the EWS results in pushing conformation of the molecule from “closed” to “open” state, making it accessible for other molecules, such as subunits of RNA Pol II. Following this mechanism, the subunit Rpb3 from Pol II is interacting weakly with EWS that induces changes in the conformation of the molecule, leaving its N-terminal part accessible for interaction with Rpb5 and the heterodimer Rpb4/7. (c) Mechanism II. An intramolecular interaction inside the EWS results in pushing conformation of the molecule from “closed” to “open” state, making it accessible for other molecules, such as subunits of RNA Pol II. Following this proposed mechanism, the interacting partners pushing the conformation of EWS from “closed” to “open” state are only the subunit of RNA Pol II Rpb3 and the complex Rpb4/7.

Thus, two independent mechanisms of transition can be described, leading to changes in the conformation of the molecules and making them accessible to interact each with other and with other molecules (Figures 2(a), 2(b), and 2(c)).

*Mechanism I*. A molecular interaction between two EWS molecules arises, pushing conformation of the molecule from “closed” to “open” state (Figure 2(a)). The EWS-EWS intermolecular protein-protein interaction induces the transition from “closed” to “open” conformation of the molecule, thus making the N-terminal part of EWS accessible for interaction with Pol II subunit Rpb5 and the heterodimer Rpb4/7.

*Mechanism II*. An intramolecular interaction inside the protein EWS results in pushing conformation of the molecule from “closed” to “open” state, making it accessible for other molecules, such as Pol II. In these interactions two or more subunits of RNA Pol II may take part in a simultaneous or successive way. The main acting components, proposed, are the subcomplex Rpb4/7 and subunits Rpb3 and Rpb5 (Figure 2(b)). Following this mechanism, the subunit Rpb3 from Pol II is interacting weakly with protein EWS that induces changes in the conformation of the molecule, leaving its N-terminal part accessible for interaction with Rpb5 and the heterodimer Rpb4/7. Following the next proposed mechanism (Figure 2(c)), the interacting partners, pushing the conformation of protein EWS from “closed” to “open” state, are only the subunit of RNA Pol II Rpb3 and the complex Rpb4/7.

Following the reported experimental data, concerning the self-association of protein EWS [24, 83] described above, Mechanism I (Figure 2(a)) is the most likely possible mechanism, because of the proven oligomerization of protein EWS, evidently important for the functional interactions of the protein, and especially with RNA Pol II.

### 3.5. Proposed Model of the Transcription Complex

**Figure 3:**
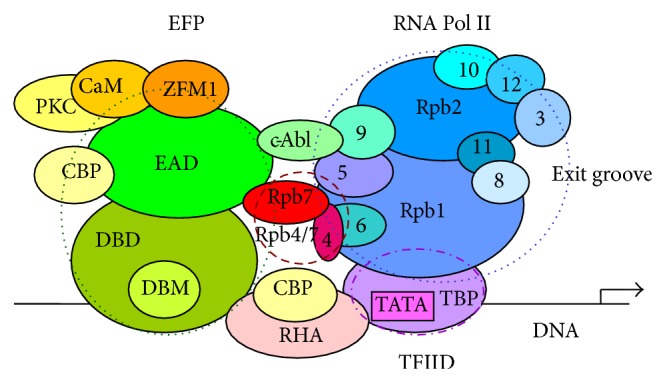
Schematic model of the transcription complex (transactivation by the EAD), including some of the interacting partners of EWS, based on the EFPs fusion proteins. The EAD is bound to the promoter via the DNA-binding motif (DBM) in the DNA-binding domain (DBD) of the EFP (bZIP for the ATF1 as EFP), and Core Pol II binds DNA via the TBP TATA box of the transcription factor TFIID. The N-terminus of EAD directly contacts Rpb5 and Rpb7 via the N-terminus, while the intact EWS does not. The heterodimer Rpb4/7 binds to the EAD, thus connecting it to the Core RNA Pol II. The complex Rpb4/7 can stabilize the pre-initiation complexes by converting the conformation of RNA Pol II from open to closed. The Rpb7 is forming several direct contacts with Rpb1, Rpb2, and Rpb6 holding them together in a preferred conformation. The conformation of Pol II changes during different stages. The Core Pol II may adopt an open configuration, allowing the dsDNA to enter the active-site groove. The Rpb4/7 associates with Core Pol II through the N-terminal ribonucleoprotein-like domain of Rpb7 and the partially ordered N-terminal region of Rpb4. Some additional components, CBP, PKC, CaM, and ZFM1, interacting with the EAD, and RHA, interacting with EFP, are shown. DBD-DNA-binding domain. DBM-DNA-binding motif; for EAD-ATF1 the DBM is bZIP.

A model of the transcription complex was made, based on the EWS/EFPs fusion proteins as shown in Figure 3. The proposed model is schematic, taking into account the possible partners and interactions of the complex, as suggested from the different experimental data. The complex formation has a dynamic character, with possible changes in the conformations of the components.

Schematic model of the transcription complex (transactivation by the EAD), including some of the interacting partners of EWS, made on the EWS/EFPs, was shown in Figure 3. The EAD is bound to the promoter via the DNA-binding domain, and Core Pol II binds DNA via the TBP TATA box of the TF TFIID. The N-terminus of EAD directly contacts Rpb5 and Rpb7 via the N-terminus, while the intact EWS does not. The heterodimer Rpb4/7 binds to the EAD, thus connecting it to the Core RNA Pol II. The complex Rpb4/7 can stabilize the pre-initiation complexes by converting the conformation of RNA Pol II from “open” state (without Rpb4/7, “Rpb4/7-free”) to “closed” state (with Rpb4/7, “Rpb4/7-bound”). The subunit Rpb7 of Pol II is forming several direct contacts with subunits Rpb1, Rpb2, and Rpb6, holding them together in a preferred conformation. The conformation of Pol II changes during different stages. The Core Pol II may adopt an “open” configuration, allowing the dsDNA to enter the active-site groove. The Rpb4/7 associates with Core Pol II through the N-terminal ribonucleoprotein-like domain of subunit Rpb7 and the partially ordered N-terminal region of Rpb4. Schematically, some additional components such as proteins CBP, PKC, CaM, and ZFM1, interacting with the EAD, are shown in Figure 3.

The Pol II subunits Rpb4 and Rpb7 bind RNA, thus stabilizing the transcription complex. The position of the heterodimer Rpb4-Rpb7 is located near groove 1, the channel for nascent RNA exit, consistently with an RNA binding. The proximity of Rpb4/7 to the flexible clamp and its influence on clamp position in low-resolution structures suggest that the heterodimer may modulate the position of this flexible module [84, 85].

A model of the functional interactions in transcription and splicing of Ewing's sarcoma is proposed in [86]. It is not clear at what stage the interacting partners are acting, their place in the process, and the mechanism of action. The role of each of them in the cancer pathways is not well defined and understood, as well as the functional relationships between them.

This model can help to clarify the role of each component in the mechanism of tumorigenesis and may be useful to design experiments to determine the nature, formation, and structure of the functional complexes. Additional experiments are necessary to determine the role and function of all components of the proposed model complexes and of the complicated interactions between them, including dsDNA, RNA Pol II, EWS, transcription factors, and other functional components.

The system of functional interactions of EAD in a complicated system of relationships of specific origin and nature, participating in different systems and processes, all together involved in tumorigenesis, possibly could be revealed. Now the information about the relationship between all compounds of the system, their structure, and function in rapport to cancerogenesis is insufficient to build a complete model. A lot of experimental and theoretical works have to be completed to determine all interacting partners implicated in cancer from different nature and structure, such as proteins (including enzymes), RNA, DNA, and other molecules, and to find the functional relationships between them.

### 3.6. IDP and EFPs Binding Molecules for Therapy

The EAD is an IDP that exhibits many features of fuzziness, with multiple aromatic side chains driving molecular recognition. Such highly versatile mode of molecular recognition offers a general conceptual framework for promiscuous target recognition by polyvalent IDPs, where the binding is driven predominantly by cation-*π* interactions between Ys and Rs [31].

Inhibiting the interaction of mutant cancer-specific TFs with the normal cellular binding partners, required for their oncogenic activity, provides a promising strategy for the development of uniquely effective, tumor-specific anticancer agents for Ewing's Tumor [54, 62]. EWS/FLI1 is a disordered protein target for anticancer therapy, since it is present only in tumor cells and is critical for the tumor. EWS/FLI1 induces large gene expression changes by causing small alterations in multiple stages of the gene regulation. EWS/FLI1 is working via multiple molecular mechanisms and its effective therapeutic targeting is difficult. A potent peptide called ESAP1 (TMRGKKKRTRAN) reduces the transcriptional activity of EWS/FLI1 and disrupts cell cycle kinetics in Ewing's Tumor cells [87]. (S)-YK-4-279 as a small molecule drug is ready for continued development towards a first-in-human, first-in-class, clinical trial. The small molecule YK-4-279 blocks RHA binding to EWS/FLI1, shows decreased cyclin D levels, induces apoptosis in ESFT cells, and reduces the growth of ESFT orthotopic xenografts [54, 88].

### 3.7. Partners Based Interactions Used for Development of Anticancer Medicines

Fusion gene transcriptional targets, downstream signaling pathways, and overexpressed growth factor receptors provide novel therapeutic targets [67]. Understanding the protein-protein interactions, protein partners, and transcriptional targets of EWS/FLI1 and the pathways that are regulated by these partnerships will inform both oncogenesis and therapeutics [55].

The inhibition of B-ZIP TFs could be therapeutically useful in cancer cells, where the oncogenesis is driven by a B-ZIP protein, such as clear cell sarcoma, with EWS/ATF1 as oncogenic fusion [45]. The residues (1–57) of EAD and hsRpb7 interact* in vitro*, indicating that DHR-related peptides and other small molecules, targeted to the N-terminus of EWS, might possess therapeutic potentialities as anticancer agents [77].

No functionally significant post-translational modifications of EWS/FLI1 have been shown. The acetylation regulates EWS/FLI1 and could be a specific target for the activity of Histone Deacetylase Inhibitors, inducing cell death of ESFT cells [38]. EWS/FLI1 may gain access to chromatin as a result of histone acetylation or undergo regulation by direct acetylation, important for treatment with HDAC inhibitors [89].

Multiple direct targets have been confirmed through demonstration of EWS/FLI1 binding to their promoters including insulin-like growth factor (IGF) binding protein 3 and the Aurora A and B kinases [67]. Neuropeptide Y (NPY) and two of its receptors, Y1R and Y5R, are upregulated by EWS/FLI1 and abundantly expressed in ES cells. NPY acting via Y1R and Y5R stimulates ES cell death and may inform novel therapeutic approaches to ES [90].

The translational potential of potent and specific LSD1 inhibition with HCI2509 on the transcriptional program of both EWS/FLI and EWS/ERG was evaluated* in vitro* and* in vivo* in xenograft models of Ewing's sarcoma. HCI2509 caused a dramatic reversal of both the up- and the downregulated transcriptional profiles of EWS/FLI and EWS/ERG accompanied by the induction of apoptosis and disruption of morphological and oncogenic phenotypes modulated by EWS/FLI. The data support epigenetic modulation with HCI2509 as a therapeutic strategy for Ewing's sarcoma and highlight a critical dual role for LSD1 in the oncogenic transcriptional activity of EWS/ETS proteins [91].

The network structure of EWS/FLI1 effects on proliferation and apoptosis shows intensive crosstalk between the pathways used for its construction. Based on the network and the transcriptome data, CUL1 was identified as a new potential target of EWS/FLI1 [57].

The upregulation of HSPA5/BIP and other chaperones indicates that YB-1 depletion may lead to induction of ER stress, possibly due to unfolded protein accumulation, or conversely that YB-1 reduces ER stress [63].

miRs with prognostic potential have been identified, and several preclinical studies suggest that miR manipulation could be therapeutically useful in this aggressive disease [69].

An imbalance between the G1 cyclin-CDK complex components and p21 (and p53 pathway) and/or p27 in Ewing's sarcoma may be responsible for uncontrolled proliferation, leading to transformation. The tumor suppressor genes Rb and p53 function by blocking entry of cells into DNA synthesis (S) phase of the cell cycle. EWS/FLI1 may affect the Rb pathway, leading to oncogenesis [56].

New EFP partners with therapeutic potential in ESFTs are expected to be discovered in the near future.

### 3.8. Future Studies

Although initial trials and* in vitro* studies have provided the way for advances in sarcoma targeted therapy, further work is needed to better characterize tumors at the molecular genetic level to tailor therapies to individual tumors [67]. Further studies are needed to identify whether EWS- or RNA-binding affinity and pre-mRNA regulation are affected in the ALS-related mutants [10]. The potential acetylation of EWS/FLI1 could have an impact on tumor response and need to be revealed [89]. Further work is required to clarify the role of YB-1 as a stress adaptor protein and its role in regulating ER homeostasis [63].

The future studies of miR biology will expand our understanding of Ewing's sarcoma pathogenesis and may identify new biomarkers and treatment options. It will be of interest to determine how miR expression and function differ in the context of the other, less common EWS/ETS fusions, as well as the more divergent non-EWS/ETS fusions [16, 69]. COX-2 expression in Ewing's sarcoma may not be directly related to mRNA stabilization by HuR. However, a correlation between COX-2 expression and nuclear HuR expression through indirect mRNA stabilization can be suggested for future studies [91].

A very important point is to find the functional consequences of EWS and EWS/FLI1 homo- and hetero-dimerization on RNA transcription and splicing. The further research could continue in the direction of finding new interacting partners of EWS and of EFPs* in vitro* and* in vivo*. From the mechanism and models interactions with high probability between the full-length EWS (or CTD) and Rpb3, between the EAD (NTD fragments) and Rpb5, and between the EAD and the heterodimer complex Rpb4/7 could be predicted. The proposed interactions may be dynamic, lasting very limited time to be detected* in vitro* because of the high intrinsic disorder of EWS. They could also be a question of protein organization, mobility, and flexibility because of the intrinsic disorder of the interacting partners, predominantly TFs. Inhibiting these interactions, required for the oncogenic activity, may help in the development of uniquely effective, tumor-specific anticancer agents for Ewing's Tumor.

## 4. Conclusions

The N-terminal part of EAD is responsible for the functional interaction with RNA Pol II through the subunit hsRpb7. The conserved tyrosine residues in DHRs can be required for interaction between the EAD and Rpb7. The observed specific interaction strongly suggests the therapeutic potentialities of anticancer agents targeted against the N-terminal part of EAD that is critical for transactivation and might function as inhibitors of the EAD-mediated transactivation. The functional interaction between the EAD and hsRpb7 may help to design and test peptides and other small molecules, including DHR-related peptides for immunogenity. The proposed model helps to understand the heterodimer Rpb7/Rpb4 function and the transcriptional mechanisms in human. The partnership between EAD and RNA Pol II poses principal questions about the functional molecular interactions in cancer and could lead to new findings, including new partners and components of the complex, to understand the mechanism of cancerogenesis and find new ways to treat cancer.

## Figures and Tables

**Table 1:** Cancer-associated gene fusions in ESFTs and EWS Fusion Proteins (EFPs) related tumours. Presented are the Fusion genes (providing DNA-binding) of EAD (providing Trans-activation) in human cancer. EFPs and the tumors that they cause: Ewing's sarcoma, Desmoplastic Small Round Cell Tumor, Myxoid Liposarcoma, Extraskeletal Myxoid Chondrosarcoma, Malignant Melanoma of Soft Parts or Clear Cell Sarcoma, Acute leukaemia. The EAD contains a similar region of EWS in each case, including minimally the first seven exons of EWS. The EWS homologue translocated in Liposarcoma (TLS) provides the EAD. EWS fusion partners provide the DNA-binding domain of the EFP.

Trans-activation	DNA-binding	Tumor	Karyotype
EAD	FLI-1	Ewing's sarcoma family of tumours	t(11;22)(q24;q12)
EAD	ERG	Ewing's sarcoma family of tumours	t(21;22)(q22;q12)
EAD	ETV1	Ewing's sarcoma family of tumours	t(7;22)(p22;q12)
EAD	ETV4/E1AF	Ewing's sarcoma family of tumours	t(17;22)(q12;q12)
EAD	FEV	Ewing's sarcoma family of tumours	t(2;22)(q33;q12)
EAD (TLS)	CHOP	Myxoid liposarcoma	t(12;22)(q13;q12)
EAD	ATF-1	Malignant melanoma of soft parts/soft tissue clear cell sarcoma	t(12;22)(q13;q12)
EAD	WT1	Desmoplastic small round cell tumour	t(11;22)(p13;q12)
EAD	ZSG	Small round cell sarcoma Ewing's sarcoma/PNET (rare)	t(1;22)(p36.1;q12)
EAD	POU5F1 (OCT3/4)	Undifferentiated bone sarcoma	t(6;22)(p21;q12)
EAD	NR4A3 (CHN/TEC or NOR1)	Extraskeletal myxoid chondrosarcoma	t(9;22)(q22;q12)
EAD	CIZ/NMP4 ZNF384	Acute leukaemia	t(12;22)(p13;q12)
FUS (TLS)	CHOP	myxoid liposarcoma and round cell liposarcoma	t(12;16)(q13;p11)
EWSR1	ATF1/CREB1	Angiomatoid fibrous histiocytoma	t(2;22)(q33;q12)
EWSR1	ERG	The Ewing sarcoma (ES) family of tumors	t(1;21;7)(q25;q22.3;q22)
EWSR1	CREB1	Angiomatoid fibrous histiocytoma clear cell sarcoma	t(2;22)(q34;q12)
RBP56/hTAFII6	CHN orphan nuclear receptor	Extraskeletal myxoid chondrosarcomas	t(9;17)(q22;q11.2)
EWSR1	hSNF5/INI1 (SMARCB1)	Extrarenal rhabdoid tumors of soft tissue/cell soft tissue sarcomas	t(1;11)(q12;q25)
EWSR1 or Not identified	Not identified or Fli-1	EWS/PNET Soft tissue sarcoma	t(18;19)(q23;q13.2)
EWS	UQCRH	small round cell sarcoma	t(1;22)(p34;q12)
EWSR1	NFATC2	(ES/PNET)	t(20;22)(q13;q12)

## Abbreviations

Pol II: RNA polymerase II
NTD: N-terminal domain
CTD: C-terminal domain
EWS: Ewing's sarcoma protein
EFP: EWS fusion proteins
EAD: EWS-Activation-Domain
IDPs: Intrinsically disordered proteins
IDRs: Intrinsically disordered regions
RHA: RNA helicase A
ESFT: Ewing's sarcoma family of tumors
FETs: FUS, EWS, and TAF15 proteins
TFs: Transcription Factors
TAD: Transcriptional activation domain
DBD: DNA-binding domain
TBP: Translocation breakpoint
Fli1: Friend leukemia integration 1 transcription factor
ATF1: Cyclic AMP-dependent transcription factor ATF-1
AA: Amino acid.
